# Does realizing strengths, insight, and behavioral practice through a psychological intervention promote personality change? An intensive longitudinal study

**DOI:** 10.1177/08902070231225803

**Published:** 2024-01-15

**Authors:** Mathias Allemand, Gabriel Olaru, Mirjam Stieger, Christoph Flückiger

**Affiliations:** 1 27217 University Research Priority Program “Dynamics of Healthy Aging,” University of Zurich, Zurich, Switzerland; 2 7899 Department of Developmental Psychology, Tilburg University, Tilburg, Netherlands; 3 Institute of Communication and Marketing, Lucerne University of Applied Sciences and Arts, Lucerne, Switzerland; 4 9178 Department of Psychology, University of Kassel, Kassel, Germany

**Keywords:** personality states, generic change factors, digital personality change intervention, random intercept cross-lagged panel model

## Abstract

The mechanisms of change underlying the effectiveness of personality change interventions are largely unclear. In this study, we used data from a three-month digital intervention with an intensive longitudinal design to test whether a greater realization of general change factors is partly responsible for personality change. Participants (*N* = 679, 53.0% female; age: *M* = 25.3 years, *SD* = 7.1) seeking to increase either Emotional Stability, Conscientiousness, or Extraversion provided self-ratings on their weekly personality states and the three generic change factors of strengths, insights, and behavioral practice. We found a single-factor structure of change factors within and between individuals. Results showed within-person increases in Emotional Stability, Extraversion, and Conscientiousness states as well as increases in change factors across the intervention. Changes in personality states were coupled with changes in generic change factors. Finally, the results provide initial support for the hypothesis that the realization of general change factors is partly responsible for the effects of the intervention. Within-person increases in the change factors were associated with subsequent increases in Extraversion and Emotional Stability states during the following week. The present findings highlight the need to better understand how and why people change in personality as a result of interventions.

## Plain language summary

Recent studies have shown that it is possible to change personality traits through psychological interventions. However, the mechanisms of change are largely unclear. The present study therefore examined whether targeting supportive (strengths), learning (insight) and action (behavioral practice) factors through intervention contributes to personality change. Our results provide initial evidence that these general change mechanisms are partly responsible for personality changes.

Personality traits are typically defined as relatively stable patterns of behavior and experience (Baumert et al., 2017). Recent conceptual work challenges this relative stability and discusses conceptual approaches to trait change through interventions (e.g., Allemand & Flückiger, 2017; Rebele et al., 2021; Roberts, Hill, & Davis, 2017). Moreover, recent findings from intervention studies suggest that trait change through interventions is possible and that traits can be changed relatively quickly over a short period of time through clinical and non-clinical interventions (e.g., Jackson et al., 2021; Roberts, Luo, et al., 2017; Stieger et al., 2021; Stieger, Wepfer, et al., 2020). These initial conceptual accounts and empirical results are promising and highlight the potential for intervention efforts. So far, however, it is largely unclear which mechanisms are responsible for the change. One conceptual framework—the generic change factors (GCF) model (Allemand & Flückiger, 2017, 2022)—suggests that realizing general factors such as strengths, insight, and behavioral practice maximizes intervention effects. So far, this assumption has not been explicitly tested. The present study therefore examined whether individual changes in weekly Big Five personality-related behaviors, thoughts, and feelings, referred to as weekly personality states, during a personality intervention are concurrently and prospectively associated with the strength of general change factors.

## Conceptual models for personality change interventions

Existing conceptual work discusses various intervention models in terms of specific change processes and intervention approaches. For example, one framework focuses on behavioral activation to increase Conscientiousness (Magidson et al., 2014; Roberts, Hill, & Davis, 2017). The main goal is to increase engagement in goal-directed activities that are considered important, enjoyable, and in accordance with individual values and goals across numerous domains of life. In terms of an intervention to change personality traits, this means that the intervention primarily requires individuals to increase the expression of traits by regularly practicing new personality-related behaviors and experiences, which then form new habits and eventually solidify into more permanent trait change. This idea is consistent with recent theoretical models of personality development that emphasize bottom-up processes that can accumulate into long-term personality changes (Allemand & Flückiger, 2017; Chapman et al., 2014; Wrzus & Roberts, 2017). Other conceptual accounts suggest behavioral and cognitive-behavioral interventional pathways to increase Conscientiousness (Javaras et al., 2019) or decrease Negative Emotionality (Sauer-Zavala et al., 2017). For example, cognitive-behavioral techniques, such as mindfulness techniques, can be used to help people respond more flexibly to stressful situations and events that trigger neurotic thoughts and feelings, rather than automatically responding to stressful situations with avoidance responses (Armstrong & Rimes, 2016).

Other researchers focus on self-regulation processes as potential intervention targets (Hennecke et al., 2014; Nevins, 2021; Rebele et al., 2021), and suggest intervening on short-term (e.g., goal activation and selection or modification of situational features) and long-term self-regulation processes (e.g., behavior repetition for habit formation). Finally, one of the first conceptual work on intentional personality change proposes a stepwise process model of coaching that includes 10 specific steps (Martin et al., 2014): Assess personality and clients values; discover the present self; explore gaps between actual self and desired self; choose personality facet change goals; assess attitudes toward change; design and implement coaching plan; re-assess personality and review progress; implement remaining coaching sessions; re-assess, review and maintain; follow-up, review and refinement. This conceptual framework is closely related to behavioral and cognitive-behavioral models from the coaching literature (Eldridge & Dembkowski, 2013; Palmer & Williams, 2013), and provides concrete guidelines for intervention techniques and activities for each step of the model.

## Strengths, insight, and behavioral practice as generic change factors

The generic change factors (GCF) model (Allemand & Flückiger, 2017), inspired by the clinical and psychotherapy process-outcome literature (Grawe, 2004; Lambert & Ogles, 2004; Norcross & Goldfried, 2019; Wampold & Imel, 2015; see also Castonguay et al., 2015; Lutz et al., 2021 for critical reviews), takes a different conceptual approach. Although personality change interventions may involve a number of specific intervention goals, techniques, and activities, the GCF model assumes that the intervention should be largely collaborative and based on general factors or principles. In fact, several general factors are discussed in the psychotherapy literature (e.g., working alliance; e.g., Wampold & Flückiger, 2023), which can be grouped into three broad categories of support, learning, and action (see Cuijpers et al., 2019; Lambert & Ogles, 2004 for details). Supportive factors are believed to facilitate learning experiences and explorative behaviors. Learning factors refer to self-reflection processes that are triggered and maintained by interventions. Action factors include exploration and the repeated performance of (new) behaviors. The GCF model was adapted from a framework consisting of four empirically derived general change mechanisms based on a comprehensive meta-analytic review of results from controlled psychotherapy studies and naturalistic process-outcome studies (Grawe, 2004; Grawe et al., 1994; Orlinsky et al., 1994). The adaptation was primarily motivated by the goal of providing some heuristic principles for the development and implementation of personality change interventions in normal populations that do not particularly suffer from mental disorders (Allemand & Flückiger, 2017). Specifically, the GCF model suggests that personality change can be achieved through the realization of four general change factors: Discrepancy awareness, strengths, insight, and behavioral practice (Allemand & Flückiger, 2017, 2022).

First, actuating *discrepancy awareness* is a support factor. By exploring possible differences between desired and actual personalities and by explicitly activating discrepancy awareness during interventions, individuals may be more motivated to invest in change efforts. This is broadly consistent with the third step of the stepwise process model (Martin et al., 2014), which proposes that after the assessment and discovery of the actual personality, the gaps between the actual and desired personalities should be explored. Discrepancy awareness is also consistent with the cybernetic model of self-regulation (Carver & Scheier, 1998), according to which one has to realize a difference between the current self and a “standard” or “ideal self” in order to initiate self-regulation. It also addresses one important condition for self-regulated personality development (Hennecke et al., 2014), namely, the desirability of change. Activating a discrepancy awareness can lead to perceiving change as desirable.

Second, activating people’s *strengths* and resources can serve as a support factor for initiating and maintaining change processes. This can be done by capitalizing on existing personality traits, motivations, skills, interests, and social relationships as strengths and resources (Flückiger et al., 2023). It is believed that activating strengths initiates and maintains positive feedback loops and expectations, thus having a beneficial effect on change efforts. This support factor addresses another important condition for self-regulated personality development (Hennecke et al., 2014), namely, the feasibility of change. Activating strengths can promote perceptions of feasibility and shift the focus from difficulties and challenges to feasibility.

Third, promoting *insight* is an important learning factor. It focuses on change processes through self-reflection on behaviors and experiences. Interventions should therefore target reflective processes that help people to better understand their assumptions, expectations, and motivations and to make new connections about themselves, others, and experiences. This learning factor reflects one comprehensive pathway in which repeated short-term situational processes can manifest into long-term personality development, as proposed by the TESSERA (Triggering situations, Expectancy, States/State Expressions, and ReActions) framework (Wrzus & Roberts, 2017). According to the TESSERA framework, one specific pathway in which short-term processes can lead to personality change is through reflective processes that involve consciously thinking and talking about past experiences, behaviors, thoughts, and feelings.

Fourth, promoting *behavioral practice* is an action factor. It refers to learning and practicing (new or modified) behaviors and skills (e.g., compensatory and coping skills), and exploring behavioral expressions in new social roles. Interventions should therefore target behaviors to help people explore and practice new behaviors and gradually increase their engagement in new activities and behaviors outside their “comfort zone.” This action factor addresses the third requirement for self-regulated personality development (Hennecke et al., 2014), namely, that change should become habitual. Through constant practice, behaviors can become habits and eventually solidify into a more enduring trait change. Another comprehensive pathway in the TESSERA framework (Wrzus & Roberts, 2017), which is partly similar to the action factor, involves associative processes. These processes include habit formation/change as implicit learning from repeated behavior, reinforcement learning through pleasant or unpleasant reactions from others, or model learning through imitation of other behaviors and experiences.

Because the field of personality change interventions is still in its infancy and the mechanisms of change are largely unclear, the GCF model provides useful heuristic principles for intervention research. For example, depending on this model, different intervention routes and conditions can be contrasted, such as learning-related versus action-related intervention strategies (Allemand & Flückiger, 2020; Gómez-Penedo et al., 2023; Grosse Holtforth & Flückiger, 2012), or strengths-related versus problem-related intervention strategies (Allemand et al., 2022; Cheavens et al., 2012; Flückiger et al., 2021), to examine the unique effects of intervention routes based on individual general factors. Moreover, a key assumption of the GCF model is that realizing all factors should maximize intervention effects (Allemand & Flückiger, 2017, 2022). This assumption guided the development and implementation of interventions targeting personality facets such as self-discipline or openness to action (Stieger, Wepfer, et al., 2020) or the Big Five personality traits (Stieger et al., 2021). Although these studies have implicitly assumed that the realization of change mechanisms is partly responsible for the effect of personality change interventions, this assumption has not yet been explicitly tested.

## Current evidence from personality change interventions

Numerous developmental studies have shown that despite the relative stability of personality traits, they are malleable and continue to change in adulthood and old age (e.g., Bleidorn et al., 2022; Graham et al., 2020; Olaru & Allemand, 2022; Roberts et al., 2006). However, compared to the large body of developmental research on personality traits across the lifespan, relatively little is known about *intentional* or *volitional* change through interventions. In fact, intervention research is rather new to personality science, as personality traits are rarely considered as targets of interventions. However, there is a growing body of evidence from clinical and psychotherapeutic research showing that clinical interventions in which (nonpathological) personality traits were not the direct target of the interventions may also promote personality change (e.g., De Fruyt et al., 2006; Sauer-Zavala et al., 2021; Stieger et al., 2022; Tang et al., 2009). For example, a meta-analytic review of 207 clinical intervention studies found decreases in Neuroticism (Negative Emotionality) and increases in Extraversion (Roberts, Luo, et al., 2017). Interestingly, remarkable trait changes were already evident from an intervention duration of more than 4 weeks.

Preliminary evidence for personality trait changes through intervention also comes from a few studies in non-clinical populations. Those studies used a variety of approaches, including goal setting and action planning (Hudson et al., 2020), behavioral activation (Massey-Abernathy & Robinson, 2021), engagement in behavioral activities and challenges (Hudson, 2023; Hudson et al., 2019), social skills training (Allemand et al., 2022), cognitive training (Jackson, Hill, et al., 2012), arts education (Grosz et al., 2022), structured coaching programs (Allan et al., 2018), and digital coaching interventions (Allemand & Flückiger, 2022; Allemand & Stieger, 2024). For example, recent intervention research using the GCF model explored whether Self-discipline, a facet of Conscientiousness, or Openness to action, a facet of Openness to experience, can be changed with the help of an intensive two-week digital coaching intervention with text messaging (Stieger, Wepfer, et al., 2020). Results of two studies (total *N* = 255) showed that it is possible to initiate change processes with short but intensive interventions. People who chose the Self-discipline intervention showed greater increases in Self-discipline, and people who chose the Openness to action intervention showed greater increases in Openness to action compared with the other group.

## The personality coaching intervention

A recent personality trait change intervention based on the GCF model (Stieger et al., 2021) used a digital coaching approach for intervention efforts. The digital coaching intervention was thought to guide and encourage people in their self-change efforts to increase or decrease one of the Big Five traits. The intervention was conducted using the smartphone application PEACH (Android and iOS), a digital coach that automatically supports people in achieving their personality change goals (Stieger et al., 2018). During the three months of coaching individuals interacted with a chatbot twice a day and receive education, behavioral tasks, feedback, encouragement, and support. The PEACH app automatically delivers small interventions—simple techniques that help people change their thoughts, feelings, and behaviors in everyday life and trigger change processes (see also Allemand & Stieger, 2024).

The effectiveness of the digital coaching intervention was tested in a randomized controlled trial in a large sample of adults (*N* = 1523) (Stieger et al., 2021). The most frequently selected change goals were decreases in Negative Emotionality (26.7%), increases in Conscientiousness (26.1%), and increases in Extraversion (24.6%) (Stieger, Eck, et al., 2020). Outcome results based on data from the pretest, posttest, and three-month follow-up showed that participants who received the coaching reported greater changes in the particular trait they wanted to change than those in the control group (who waited one month before the coaching began) (Stieger et al., 2021). The changes were in line with the self-chosen goals for personality change and changed accordingly in the desired direction. Observers, such as friends, family members, or intimate partners, also detected significant, albeit smaller changes in participants desiring an increase but not in those desiring a decrease on a Big Five trait. A secondary data analysis examined the effects of the digital intervention at the levels of facets and nuances and found considerable heterogeneity in the intervention-related personality trait changes, most notably relatively stronger increases in Sociability (Extraversion) and weaker increases in Responsibility (Conscientiousness) related to other facets of Extraversion and Conscientiousness (Olaru et al., 2022). Finally, a recent one-year follow-up study provided initial evidence that the changes achieved by the intervention were sustained or even continued, but also highlighted the problem of attrition, which is particularly common in digital intervention studies without face-to-face contact with participants (Stieger, Flückiger, & Allemand, 2023).

To summarize, the results of the few available intervention studies suggest that personality traits can be changed, or at least processes of change can be initiated, through a variety of intervention approaches. However, the mechanisms of change are largely unclear.

## The present study

The main goal of this intensive longitudinal process study was to test the assumption that the realization of generic change factors predicts subsequent personality change (Allemand & Flückiger, 2017, 2022). Specifically, we explored whether a greater realization of strengths, insight, and behavioral practice through an intervention would be concurrently and prospectively associated with stronger changes in weekly personality states. We used process data from an existing personality coaching intervention (Stieger et al., 2021) and focused on participants with the goal of increasing Emotional Stability (i.e., reducing Negative Emotionality), Extraversion, or Conscientiousness (Stieger, Eck, et al., 2020). We also focused on the GCF of strengths, insight, and behavioral practice, as discrepancy awareness was not included in the measure of generic change factors.

We had four specific research goals: First, we examined the factor structure of the GCF measure, both at the between- and within-person level. The goal was to examine whether individuals would focus on different GCF separately or simultaneously. Second, we examined the within-person change trajectories of weekly personality states and the GCF across the intervention. In accordance with the outcome-related research on personality trait change based on pretest, posttest, and 3-month follow-up results (Stieger et al., 2021), we expected an increase in the states of Emotional Stability, Conscientiousness and Extraversion in the corresponding change goal group. Third, we examined the concurrent within-person associations between changes in the weekly personality states and the GCF. We expected that stronger increases in the change factors are associated with stronger increases in personality states. Fourth, we sought to explore bidirectional prospective associations between the weekly personality states and GCF. We expected that increases in the GCF were associated with increases in the personality state level in the following week, and vice versa.

## Method

We used intensive longitudinal process data from a digital personality coaching intervention (Stieger et al., 2021)^1^. The analyses were exploratory and not pre-registered. Data, analysis code, and research materials can be found on the Open Science Framework (https://osf.io/kf6sr). The original study obtained ethical approval by the Ethics Committee of the Philosophical Faculty of the University of Zurich (No. 17.8.4; Date of approval: August 31, 2017).

### Procedure

German-speaking adults from Switzerland were recruited. To be eligible for the study, participants had to be: 18 years or older; able to read German; owner of a smartphone (Android or iOS) with mobile Internet connection; motivated to change one personality trait; not in a psychotherapeutic or psychiatric treatment; and pass an online mental health screening, as the intervention focused explicitly on healthy adults.^2^ University mailings and social media advertisements were used for the recruitment process. Additionally, potential participants responded to flyers or word-of-mouth recruitment. Interested people were directed to either the website of the project or to the Apple Store/Google Play Store to receive detailed information about the study aims, interventions, assessments, reimbursement, and data protection and to download the mobile application.

At the beginning of the study, participants were asked to choose one out of nine change goals (i.e., a combination of the Big Five trait domains and increase vs. decrease; except for decrease in Emotional Stability) from a list describing each change goal (see Stieger, Eck, et al., 2020 for details). Participants were asked to provide responses to the outcome measures (i.e., personality states and generic change factors) at the end of each week from the 1^st^ to the 11^th^ week of the intervention (i.e., the week before the end of the intervention).

### Participants

Of the 1523 participants who joined the intervention, 874 provided at least one weekly assessment. Of these, most wanted to increase Emotional Stability (*n* = 257), Conscientiousness (*n* = 226), or Extraversion (*n* = 196). The next largest groups wanted to increase Openness (*n* = 63) or decrease Agreeableness (*n* = 57). To have a sufficiently large power and sample size for the model estimation within each group, we only focused on the three largest change goal groups. The 679 participants (*n* = 360/53.0% female) were on average 25.3 years old (*SD* = 7.1). An overview of the number of participants at each wave and the number of repeated measures for each group is given in Table 1.

**Table 1.** Number of participants per group, wave, and number of repeated measures.

Number of participants at each wave											
Week	1	2	3	4	5	6	7	8	9	10	11
ES group	246	205	183	156	140	125	118	108	102	98	105
CO group	215	170	140	119	103	96	88	79	73	70	84
EX group	185	142	117	99	91	84	76	68	52	53	65
Total	646	517	440	374	334	305	282	255	227	221	254
Number of repeated measures for each group											
Number of waves	1	2	3	4	5	6	7	8	9	10	11
ES group	42	23	25	25	17	5	7	13	10	25	65
CO group	55	29	16	15	12	10	8	5	8	20	48
EX group	50	27	12	8	15	8	8	9	12	16	31
Total	147	79	53	48	44	23	23	27	30	61	144

*Note.* ES: emotional stability; CO: conscientiousness; EX: extraversion.

With respect to the highest level of education participants, most participants from the three largest intervention groups had a general qualification for university entrance (46.5%), 21.2% had a Bachelor’s degree, 16.8% a Master’s degree, 6.2% completed vocational training and education, 3.7% were secondary school graduates, and 1.8% had a PhD. We did not assess income in this study, but 54.3% of the participants were students, 20.9% working full-time, 22.1% working part-time, 0.1% home-maker, 0.4% retired, and 2.1% were currently not working. With respect to relationship status, 55.2% were currently in a relationship or married, 41.1% were single, 0.8% were separated or divorced, and 2.8% did not want to answer this question. Of the sample, 36.1% lived with their parents, 30.2% lived in a shared apartment, 21.4% lived with their partner, 11.9% lived alone, and 0.4% lived with their children but without a partner. Most participants did not have any children (93.2%). We did not assess ethnicity in our study, but 90.9% of participants indicated that their mother tongue was German, 82.3% that their nationality was Swiss, and 11.2% that their nationality was German (6.4% reported other nationalities).

### Measures

#### Weakly Big Five states

At the end of each week, participants were asked to fill out the German Big Five Inventory-2 short version (BFI-2S; Danner et al., 2019; Soto & John, 2017a, 2017b). The BFI-2S is a 30-item measure of the Big Five personality traits. To focus on participants’ personality states during the last week, we adjusted the instruction (i.e., “Please rate the extent to which the following statements apply to yourself *in the last week*”) and items (e.g., “I *was* rather quiet”). Participants responded to the items on a 5-point Likert scale ranging from 1 *= strongly disagree* to *5 = strongly agree*. We focused on the three domains Emotional Stability (i.e., reverse coded Negative Emotionality), Extraversion, and Conscientiousness in line with the three intervention groups. The average Cronbach’s alpha across the 11 measurement occasions was α = .84 for Emotional Stability, α = .77 for Conscientiousness, and α = .68 for Extraversion.

#### Generic change factors

At the end of each week, participants were asked to assess their change progress based on three GCF. To assess *strengths* activation, we developed a 3-item measure. The items were inspired by measures of resource activation from psychotherapy process research (Flückiger et al., 2010; Trösken, 2016). To assess *insight* and *behavioral practice*, we adapted a 6-item measure from psychotherapy process research that was developed to measure therapy progress after therapy sessions and to determine whether the client better understands and has insight into their problems and is now better able to cope with their own problems. The measure was developed and modified by multiple research groups (e.g., Flückiger et al., 2010; Krampen & Wald, 2001; Schulte, 2005). All items are shown in Table 2. To focus on GCF during the last week, we adjusted the instruction (i.e., “Please answer the following questions *related to last week*”). Participants responded to all 9 items on a 7-point Likert scale ranging from 0 *= not at all* to 6 *= yes exactly* with a center point of *3 = neither nor*. Across the 11 measurement occasions, the average Cronbach’s alpha value was α = .82 for strengths, α = .82 for insight, and α = .83 for behavioral practice (α = .92 for the total score).

**Table 2.** Items of the measure of generic change factors.

Item	Original item in German	English translation	Subscale
1	Diese Woche habe ich mehr an Verständnis und Einsicht in meine Erlebens- und Verhaltensweisen gewonnen.	This week I have gained more understanding and insight into my experience and behavior.	Insight
2	Ich glaube, dass ich mich jetzt besser so verhalten kann, wie ich möchte.	I believe that I can now behave better the way I want to.	Practice
3	Meine Stärken und Fähigkeiten sind mir nach dieser Woche deutlicher geworden.	My strengths and abilities have become clearer to me after this week.	Strengths
4	Nach dieser Woche sind mir Zusammenhänge klar geworden, die ich bisher nicht gesehen habe.	After this week, I have become aware of connections that I did not see before.	Insight
5	Nach dieser Woche traue ich mir mehr zu, meine Erlebens- und Verhaltensweisen aus eigener Kraft zu verändern.	After this week, I have more confidence in myself to change my experiences and behavior by my own efforts.	Practice
6	Nach dieser Woche sehe ich, dass es viele Dinge in meinem Leben gibt, die mir Kraft und Zuversicht geben.	After this week, I see that there are many things in my life that give me strength and confidence.	Strengths
7	Nach dieser Woche sehe ich bestimmte Dinge in neuem Licht.	After this week, I see certain things in a new light.	Insight
8	Nach dieser Woche fühle ich mich Situationen besser gewachsen, denen ich mich bisher nicht gewachsen gefühlt habe.	After this week, I feel more able to cope with situations that I did not feel able to cope with before.	Practice
9	In der letzten Woche sind mir viele positive Seiten an mir und meinem Leben bewusst geworden.	During the last week I have become aware of many positive aspects of myself and my life.	Strengths

*Note.* The English translation reflects a nonprofessional translation with an online translator. The sources of the items are given in the text.

### Statistical analyses

The multi-level confirmatory factor analysis was run in Mplus 8.0 (Muthen & Muthen, 2016). All other analyses were run in R version 4.1.3 (R Core Team, 2022) with the R packages *ggplot2* (Wickham, 2016), *haven* (Wickham & Miller, 2020), *lavaan* (Rosseel; 2012), and *psych* (Revelle, 2020).

#### Multi-level confirmatory factor analysis

To answer the first research question (i.e., what is the structure of the GCF?) we used a multi-level confirmatory factor analysis. We compared a model with the assumed 3-factor structure with the factors of strengths, insight, and behavioral practice at both the between- and within-person level to an alternative model with only one factor at both levels. Model fit was assessed using three approximate fit indices in line with previous guidelines (Bentler, 1990; Hu & Bentler, 1999): the *comparative fit index* (CFI; >.90 acceptable, >.95 good), *root mean square error approximation*, and *standardized root mean square residual* (RMSEA, SRMR; <.08 acceptable, <.05 good). We then compared the models based on model fit (i.e., AIC, BIC, CFI, RMSEA, and SRMR) and factor loadings and correlations. Models were estimated with full information maximum likelihood estimation (FIML) to account for missing values.

#### Latent growth curve model

To answer the second research question (i.e., do personality state and GCF levels change across the course of the intervention?) we used a latent growth curve model. We specified a level (or intercept) factor loading on each weekly state or generic change factors score with λ = 1, and a linear change (or slope) factor loading on each weekly state or GCF score with increasing loadings across time (i.e., λ = 0 to 10). To test whether change would be better described by a curve-linear function, we also tested a model with an additional quadratic slope factor centered around week 6 (i.e., λ = 25, 16, 9, 4, 1, 0, 1, 4, 9, 16, 25 from week 1 to 11). We used the same model fit cut-offs mentioned above.

#### Random intercept cross-lagged panel model

**Figure 1.**
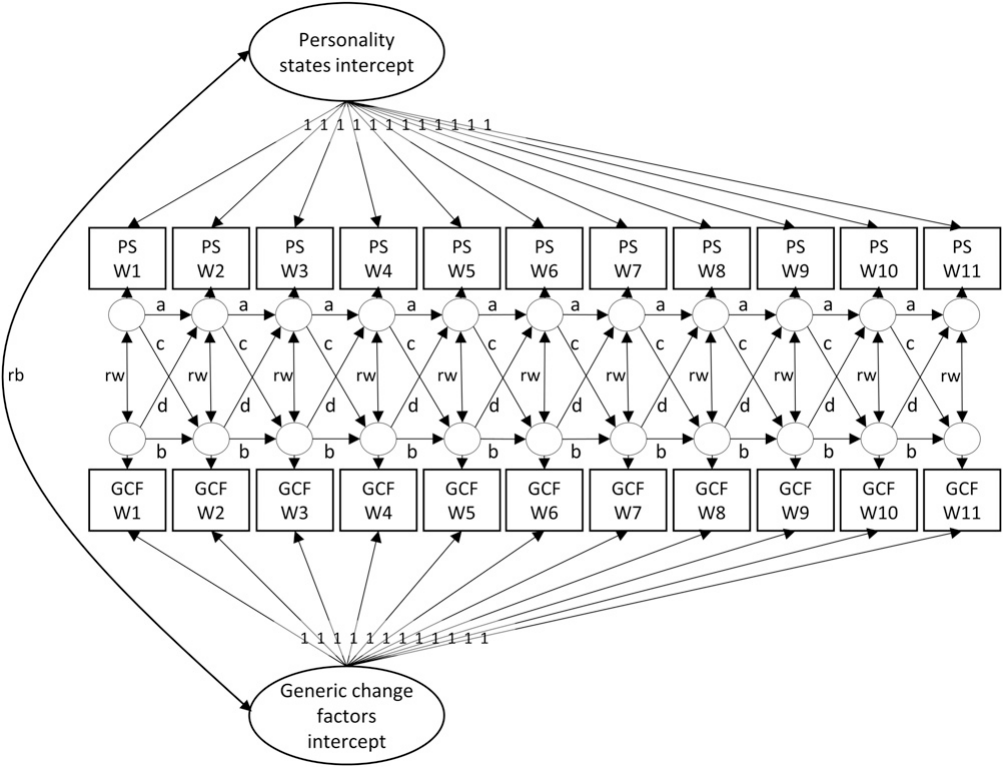
Random intercept cross-lagged panel model. *Note.* PS: personality state; GCF: generic change factor; W1–W11: week 1–week 11; a/b: autoregressive effects; c/d: cross-lagged effects; rb: between-person (random-intercept) correlation; rw: within-person (occasion-specific) correlation. Squares denote observed variables (i.e., scale scores at each measurement wave) and ellipses represent latent factors (i.e., random intercepts and wave-specific deviations).

To answer the third and fourth research question (i.e., are personality states and GCF associated within and across time?), we used a *random intercept cross-lagged panel model* (RI-CLPM; see Figure 1; Hamaker et al., 2015; Mund & Nestler, 2019). We chose this model as we were interested in the reciprocal associations between the weekly states and GCF. Specifically, we wanted to examine whether a stronger engagement in trait-consistent states would be associated with more strengths, insight, or behavioral practice in the same week (path *rw* in Figure 1), as well as the week after (path *c* in Figure 1). Similarly, we wanted to examine whether weeks in which these GCF were particularly strong would be associated with an increase in the personality-consistent states in the week after (path d in Figure 1). Compared to a traditional cross-lagged panel model (CLPM), the random intercept model accounts for stable *between-person* differences in the constructs across time. Whereas a CLPM would examine whether someone with stronger insight than other participants would show higher Extraversion states than other participants in the same week or the week after (or vice versa), the RI-CLPM focuses on *within-person* associations: For example, is a week in which a participant experiences more insight—compared to the other 10 weeks—associated with stronger Extraversion states that week and the week after (again compared to the other 10 weeks)? While we were primarily interested in the cross-lags and occasion-specific correlations, the RI-CLPM also includes autoregressive effects (path *a* and *b* in Figure 1). In a RI-CLPM these represent *carry-over* effects, or in other words how strongly the weekly states and GCF fluctuate around the stable level. For example, if the participants would engage with the intervention activities for several subsequent weeks, followed by several weeks of less engagement, the autoregressive effects should be higher. If the engagement or weekly states change from week to week, then the autoregressive effects should be lower.

We did not include additional slope factors in the model (e.g., autoregressive latent trajectory model with structured residuals; Curran et al., 2014; Mund & Nestler, 2019) as we wanted to model change on a weekly level (i.e., in the residuals). When including slope factors, the residuals reflect deviations from a (curve-) linear change, which we deemed less informative than deviations or change from a stable level. We ran this model on each change goal group separately as well as a multi-group model across all three change goal groups using the corresponding targeted weekly personality state scores. Because the associations could be constrained to equality across groups (e.g., the GCF were similarly associated with later states independent of the intervention group; see next paragraph and Table 3), we also ran the RI-CLPM on the entire dataset to increase the power to detect significant effects. Models were estimated with full information maximum likelihood to account for missing values.

**Table 3.** RI-CLPM model fit with time and group equality constraints.

Group	Time constraint	df	AIC	BIC	χ^2^	Δχ^2^	Δdf	*p*
ES	None	177	5855	6203	254			
	Autoregressive	195	5835	6119	271	17	18	.537
	Cross-lagged	213	5811	6031	282	12	18	.869
	Correlations	223	5801	5985	292	10	10	.476
CO	None	177	3903	4238	249			
	Autoregressive	195	3895	4168	276	28	18	.065
	Cross-lagged	213	3870	4082	288	12	18	.864
	**Correlations**	**223**	**3874**	**4052**	**311**	**23**	**10**	**.009**
EX	None	177	3355	3676	289			
	Autoregressive	195	3344	3606	314	25	18	.123
	Cross-lagged	213	3331	3534	337	23	18	.193
	**Correlations**	**223**	**3334**	**3505**	**360**	**23**	**10**	**.010**

Group	Group constraint	df	AIC	BIC	χ^2^	Δχ^2^	Δdf	*p*
All	None	669	13009	13714	964			
	Autoregressive	673	13008	13695	971	7	4	.127
	Cross-lagged	677	13004	13673	975	4	4	.409
	**Correlations**	**679**	**13013**	**13673**	**988**	**13**	**2**	**.002**

*Note.* ES: emotional stability; CO: conscientiousness; EX: extraversion; df: degrees of freedom; AIC: Akaike Information Criterion; BIC: Bayesian Information Criterion; Δχ^2^: chi square difference between models; *p*: chi square difference test significance value. Higher AIC and BIC values, as well as a significant χ^2^-difference test suggest a worse fit of the constrained model (i.e., unequal parameter estimates across time or groups). Significant differences are shown in bold.

##### Equality constraints across time and groups

Model parameters of the random intercept cross-lagged panel model can be constrained to equality across time to improve statistical power, estimation precision, and interpretability of the results (Orth et al., 2021). For each model, we thus tested sequentially if (a) autoregressive effects, (b) cross-lagged effects, and (c) within-time correlations could be constrained to equality across time. And finally, we also tested whether the associations were equivalent across intervention groups. Model fit of the unconstrained and constrained models are presented in Table 3. The Bayesian Information Criteria (BIC) consistently favored the constrained models. However, the Akaike Information Criterion (AIC) and χ^2^ difference test suggested unequal occasion-specific correlations across time in the Conscientiousness and Extraversion group, as well as between groups. We thus present the findings for the constrained models, followed by an examination of the differences in within-person occasion-specific correlations.

Model fit was acceptable for all RI-CLPMs with all the aforementioned equality constraints across time (ES: *df* = 223; χ^2^ = 292; CFI = .956; RMSEA = .035; SRMR = .085; CO: *df* = 223; χ^2^ = 311; CFI = .942; RMSEA = .042; SRMR = .095; multi-group model with additional group constraints: df = 681; χ^2^ = 989; CFI = .930; RMSEA = .045; SRMR = .094). The only exception was the Extraversion model (EX: *df* = 223; χ^2^ = 360; CFI = .895; RMSEA = .056; SRMR = .100), for which a larger number of small and unsystematic residual correlations caused the model fit issues (i.e., all modification indices were smaller than Δχ^2^ = 10). As such, we decided against modifying the model by incorporating these residual correlations but advise caution when examining the results.

##### Power analysis

In line with recommendations by Orth et al. (2022), we interpret cross-lags of .03 as small, .07 as moderate, and .12 as large. To determine the power of the model to detect these cross-lags based on the currently available data, we simulated data based on these effect sizes replicating the sample size and missing data structure for each change goal group. We simulated 1000 datasets for each effect size (i.e., .03, .07, .12) and change goal group (i.e., *N*s = 679 in the entire dataset, 257 in the ES group, 226 in the CO group, and 196 in the EX group) including the missing data structure of the original data. We then estimated the model with equality constraints across time on each dataset. The results of the power analysis are presented in Table 4. Power was adequate (i.e., >.80) for moderate effect sizes in the entire dataset, but only for large effects in the three intervention groups. As such, we primarily focused on the RI-CLPM findings of the entire dataset in the following but point out deviations for the single intervention groups. We tested against α = .05, but report exact *p*-values throughout.

**Table 4.** Cross-lags power analysis.

Effect size	ES group	CO group	EX group	Full sample
β = .03	.18 (.07)	.16 (.05)	.13 (.04)	.36 (.17)
β = .07	.70 (.46)	.59 (.33)	.49 (.28)	.97 (.88)
β = .12	.99 (.95)	.95 (.86)	.92 (.79)	>.99 (>.99)

*Note.* ES: emotional stability; CO: conscientiousness; EX: extraversion; presented is the percentage of significant cross-lags with a significance level of α = .05 (α = .01).

## Results

### Factor structure of the generic change factors scale

First, we examined the structure of the GCF scale at the between- and within-person level (i.e., research question 1). To do so, we ran a multi-level confirmatory factor analysis with the assumed three-factor structure and an alternative one-factor structure. The results are presented in Table 5.^3^ Despite a better fit of the three-factor model (*df* = 48; χ^2^ = 1005; CFI = .935; RMSEA = .072; SRMR_between_ = .037; SRMR_within_ = .040) compared to the one-factor model (*df* = 54; χ^2^ = 1289; CFI = .916; RMSEA = .077; SRMR_between_ = .045; SRMR_within_ = .043), the between- and within-person factor correlations of on average .93 (between) and .90 (within) suggested little differences between the GCF. Furthermore, the average factor loadings only increased from .92 to .94 (between) and .61 to .64 (within) when estimating the three-factor solution compared to a one-factor solution. As such, it seems that a single factor can adequately capture the variance in the individual GCF items.

**Table 5.** Generic change factors (GCF): Factor loadings and correlations.

Between-person level					Within-person level			
	1-factor	3-factor			1-factor	3-factor		
Item 1	.94	.94			.60	.63		
Item 4	.94	.98			.58	.63		
Item 7	.91	.96			.59	.62		
Item 2	.94		.96		.66		.68	
Item 5	.94		.96		.68		.69	
Item 8	.95		.96		.60		.61	
Item 3	.96			.98	.65			.67
Item 6	.80			.85	.58			.61
Item 9	.86			.91	.59			.62
		Factor cor. (between)			Factor cor. (within)		
		.93				.90		
		.92	.95			.87	.93	

*Note.* Presented are standardized estimates. Model fit: one-factor model: df = 54; χ^2^ = 1289; CFI = .916; RMSEA = .077; SRMR (between) = .045; SRMR (within) = .043; three-factor model: *df* = 48; χ^2^ = 1005; CFI = .935; RMSEA = .072; SRMR (between) = .037; SRMR (within) = .040.

To check whether a potential alternative structure might describe the data better, we also ran a multi-level exploratory factor analysis ranging from one to three factors in both levels (see OSF Table S1 for results). This did not suggest a different structure from the one tested in the confirmatory factor analysis though. Finally, we checked whether the factor structure of the GCF differed with respect to the change goals groups and ran a multi-level confirmatory factor analysis with the assumed three-factor structure and an alternative one-factor structure separately for each group. The results show that the structure is the same for all groups (see OSF Table S2 for results). Because of the strong factor correlations of around .90 between the three GCF, we focused on a single-factor solution in the following analyses. However, we also ran the analyses separately for the three GCF (see OSF Figures S1 and S2). Nearly all results were the same as for the single-factor solution, except for one deviation in the RI-CLPM within-person correlations. We describe this in more detail in the corresponding section.

### Retest correlations and mean-level changes in weekly personality states and generic change factors

The average one-week retest correlations in the entire dataset/corresponding change goal group were *r* = .59/.52 for Emotional Stability, *r* = .63/.64 for Conscientiousness, and *r* = .57/.53 for Extraversion (all *p* < .001). The average GCF retest correlation was *r* = .69 in the entire dataset, *r* = .62 in the ES group, *r* = .71 in the CO group, and *r* = .74 in the EX group (all *p* < .001). Overall, this suggests a substantial stability in the weekly states but most notably in the GCF as well. This implies that those people who reported benefiting from the intervention in one week also continued to do so in subsequent weeks.

**Figure 2.**
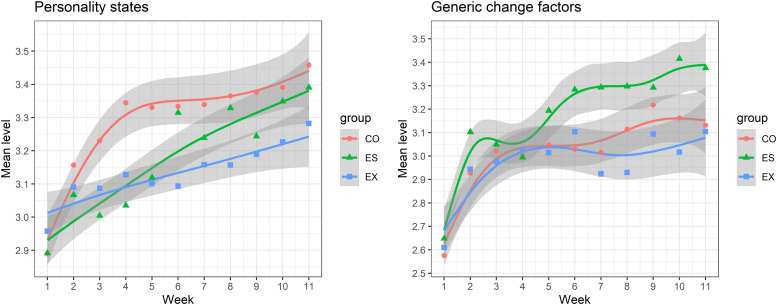
Means of weekly personality states and generic change factors across weeks. *Note*. ES: emotional stability; CO: conscientiousness; EX: extraversion; points represent the score mean in each week, lines a loess-smoothed approximation of the trend across weeks. Gray areas represent the 95% confidence intervals around the smoothed trend. For the findings for each generic change factor separately, see OSF Figure S1.

Descriptive statistics for the weekly personality states and generic change factors across time and groups are presented in Table 6. The means of personality states and change factors across time are also presented in Figure 2 (for the findings for each generic change factor separately, see OSF Figure S1). To test whether the increases were significant (i.e., second research question), we examined the mean-level changes using a latent growth curve model. AIC and BIC values and χ^2^-difference tests favored the curve-linear models, with the exception of Extraversion states in the corresponding group (see OSF Table S4). Model fit was adequate for all models, except for the Extraversion states. This suggests that the (curve-) linear slope was not a good approximation for the Extraversion change, and weekly fluctuations might be higher than for the other scores.

**Table 6.** Descriptive statistics of weekly personality states and generic change factors across weeks and groups.

ES group week	Emotional S		Conscientiousness		Extraversion		GCF	
	*M*	*SD*	*M*	*SD*	*M*	*SD*	*M*	*SD*
1	2.89	.71	3.37	.70	3.04	.61	2.65	.73
2	3.07	.75	3.52	.66	3.17	.58	3.10	.69
3	3.00	.69	3.54	.67	3.13	.53	3.05	.74
4	3.04	.76	3.52	.64	3.15	.56	2.99	.85
5	3.12	.74	3.53	.63	3.18	.57	3.19	.79
6	3.31	.75	3.63	.62	3.24	.55	3.28	.77
7	3.24	.73	3.64	.64	3.30	.57	3.29	.80
8	3.33	.78	3.63	.70	3.19	.66	3.30	.89
9	3.24	.86	3.63	.68	3.18	.59	3.29	.82
10	3.35	.77	3.74	.62	3.31	.55	3.41	.75
11	3.39	.78	3.68	.62	3.37	.60	3.38	.73

CO group week	Emotional S		Conscientiousness		Extraversion		GCF	
	*M*	*SD*	*M*	*SD*	*M*	*SD*	*M*	*SD*
1	3.43	.70	2.89	.67	3.08	.65	2.58	.71
2	3.45	.73	3.16	.62	3.25	.57	2.93	.66
3	3.35	.77	3.23	.62	3.13	.66	3.02	.70
4	3.36	.75	3.34	.65	3.15	.66	3.00	.79
5	3.40	.77	3.33	.64	3.10	.64	3.05	.77
6	3.45	.72	3.33	.62	3.23	.63	3.03	.78
7	3.49	.74	3.34	.63	3.13	.67	3.02	.82
8	3.53	.72	3.36	.71	3.16	.63	3.11	.82
9	3.57	.68	3.38	.65	3.17	.67	3.22	.85
10	3.62	.71	3.39	.63	3.24	.63	3.16	.83
11	3.55	.67	3.46	.63	3.25	.57	3.13	.86

EX group week	Emotional S		Conscientiousness		Extraversion		GCF	
	M	SD	M	SD	M	SD	M	SD
1	3.38	.72	3.40	.71	2.96	.58	2.61	.81
2	3.45	.73	3.46	.68	3.09	.64	2.94	.76
3	3.46	.73	3.52	.62	3.09	.60	2.97	.81
4	3.48	.69	3.53	.67	3.13	.54	3.03	.76
5	3.51	.61	3.60	.65	3.10	.58	3.01	.85
6	3.55	.65	3.63	.58	3.09	.59	3.10	.87
7	3.56	.66	3.56	.60	3.16	.66	2.92	.85
8	3.56	.63	3.68	.62	3.16	.61	2.93	.85
9	3.62	.74	3.65	.75	3.19	.75	3.09	.86
10	3.41	.79	3.58	.67	3.23	.58	3.02	.85
11	3.60	.72	3.63	.62	3.28	.70	3.10	.93

*Note.* ES: emotional stability; CO: conscientiousness; EX: extraversion, GCF: generic change factors.

The linear slopes indicated a weekly increase in the targeted states of on average 0.05 in the ES group, 0.04 in the CO group, and 0.03 in the EX group (all *p* < .001; values reflect unstandardized weekly changes on the 1 to 5 scale). In other words, the participants in the associated group increased by an average of 0.54 (ES), 0.42 (CO), or 0.30 (EX) across the 11 weeks covered, corresponding to a large standardized increase of *d* = 1.19 (ES), *d =* 1.03 (CO) or *d* = 0.82 (EX) (standardized based on the intercept/level factor variance). However, the significant negative quadratic effect for Conscientiousness suggested that the increase was faster at the beginning and slower towards the end of the intervention (*M* = −0.01; *p* < .001). For Emotional Stability and Extraversion, we found no significant quadratic slope. Participants differed in the rate of (linear) change, with a slope factor *SD* = 0.03 for Emotional Stability (*p* < .001), 0.04 for Conscientiousness (*p* < .001), and 0.03 for Extraversion (*p* = .002). The large majority of participants reported increases in the weekly states according to their change goal. We only found decreases in the weekly states across the intervention period for 3.5% of the ES group participants, 10.6% of the CO group, and 10.7% of the EX group.

The GCF levels increased over the duration of the intervention as well. We found an average weekly increase of 0.04 in the E–S group (*p* < .001), 0.03 in the CO group (*p* < .001), and 0.02 in the EX group (*p* = .007; values reflect unstandardized weekly changes on the 1 to 5 scale). As such, the participants showed an average increase in their insight, strengths activation, or behavioral practice by 0.45 (ES group), 0.32 (CO group), or 0.25 (EX group) across the 11 weeks covered. These increases were moderate to large in size, with standardized increases of *d* = 0.86 (ES group), *d =* 0.71 (CO group) or *d* = 0.46 (EX group) (standardized based on the intercept/level factor variance). In all three groups the curve-linear slope suggested an initially faster but subsequently slower increase (*M* = −0.01; *p* ≤ .002). Participants also differed in the rate of the (linear) change in GCF*,* with a linear slope *SD* = 0.02 for the ES group (*p* < .001), 0.03 for the CO group (*p* < .001), and 0.02 for the EX group (*p* = .018). Over the course of the intervention, majority of people reported increases in the GCF. We only found decreases for 5.4% of the ES group participants, 10.6% of the CO group, and 9.2% of the EX group.

We additionally tested whether the changes in weekly states and GCF differed across groups (e.g., whether people in the ES group showed the strongest state and intervention process changes). To do so, we compared a multi-group model with equality constraints to the slope means to a model in which they were freely estimated across groups. The AIC, BIC, and χ^2^-difference test suggested similar rates of change in the GCF across groups (see OSF Table S4). Results for the weekly states were mixed, with the AIC and χ^2^-difference test indicating significant differences (ΔAIC = 3.6; *p* = .021) in the slopes, suggesting that the Emotional Stability states increased more rapidly than the Extraversion states. However, the BIC results were in favor of the restricted model (ΔBIC = −14.5), indicating similar rates of change between change goal groups.

### Bidirectional associations between weekly personality states and generic change factors

**Figure 3.**
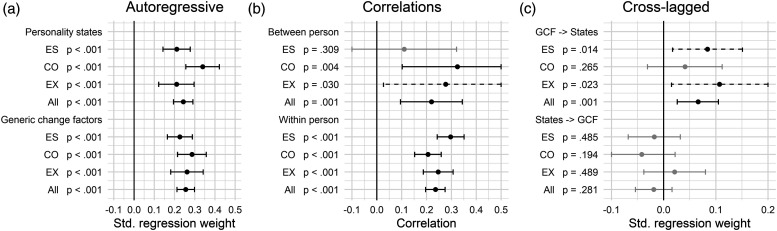
Standardized associations between weekly personality states and generic change factors. (a) Autoregressive, (b) correlations, and (c) cross-lagged. *Note*. ES: emotional stability; CO: conscientiousness; EX: extraversion; All: results from a multi-group model across all three intervention groups with equality constraints on the model parameters; Between person: random intercept correlations; Within person: occasion-specific correlations; GCF: generic change factors (i.e., strengths activation, self-insight, and behavioral practice). Presented are standardized effects with 95% confidence intervals. Gray lines indicate *p* > .05; dashed lines *p* ≤ .05; solid black lines *p* ≤ .01. For the findings for each generic change factor separately, see OSF Figure S2.

To examine the bidirectional associations between personality states and GCF across time (i.e., third and fourth research question), we ran a RI-CLPM in the full dataset and each of the intervention groups based on the combination of the targeted weekly states and change factors scores. All relevant standardized parameter estimates from the RI-CLPM analyses are presented in Figure 3 (see OSF Table S5 for exact values; estimates were standardized with the *standardizedSolution* function in *lavaan*). Unrelated to our research questions, we also present the within-person autoregressive or *carry-over* effects across weeks (Figure 3(a)). These indicated some within-person stability in the weekly states (average β = .25; all *p* < .001) and GCF (average β = .26; all *p* < .001). Because these reflect similarities in the state and GCF deviations after controlling for the stable level (i.e., between-person differences over the course of the 11 weeks), these are substantially lower than the weekly retest correlations of *r* = .59 for the states and *r* = .63 for the GCF. The within-person carry-over effects indicate that stronger deviations in the states or GCF in one week were preceded or followed by stronger deviations in the previous or subsequent weeks, indicating some form of inertia in the states and GCF across weeks.

We first wanted to examine whether those weeks in which participants reported stronger realization of GCF than usual were characterized by higher personality states in the same week (i.e., third research question). This is indicated in the occasion-specific correlations in the RI-CLPM (path *rw* in Figure 1). Results are presented in Figure 3(b). In line with our assumptions, we found that within-person changes in the weekly states were positively associated with changes in the GCF reported the same week (across all groups: *r* = .22; ES group: *r* = .30; CO group: *r* = .21; EX group: *r* = .25; all *p* < .001).

As mentioned earlier, the results using a three-factor model of GCF were very similar to the results of the single-factor model. However, there was a notable deviation in the RI-CLPM within-person correlations across all groups (see OSF Figure S2 for detailed results). *Self-insight* was only correlated by *r* = .15 (*p* < .001) with the weekly states, whereas *behavioral practice* and *strengths activation* correlated by *r* = .27 and .28 with the states, respectively (both *p* < .001).

Interestingly, the overall state and GCF levels across the 11 weeks (i.e., the random intercepts) were also positively associated (across all groups: *r* = .22; *p* = .001; no significant association for the ES group: *r* = .11, *p* = .309; CO group: *r* = .32; *p* = .004; EX group: *r* = .28; *p* = .030). Again, *self-insight* showed the weaker associations (not significant: *r* = .11; *p* = .109) compared to *behavioral practice* (*r* = .24; *p* < .001) and *strengths activation* (*r* = .28; *p* < .001; see OSF Figure S2).

And finally, we wanted to examine whether stronger realization of GCF in one week would be associated with higher personality states in the subsequent week, and vice versa (i.e., fourth research question). This is indicated in the cross-lagged effects in the RI-CLPM. The results are presented in Figure 3(c). In line with our assumptions, we found that stronger realization of GCF were associated with higher state deviations the week after (across all groups: β = .07; *p* = .001; ES group: β = .08; *p* = .014; EX group: β = .11; *p* = .015). Based on typically found effect sizes for cross-lags in such models, the effects can be considered moderate in strength (i.e., β ≥ .07; Orth et al., 2022). However, we found no significant association in the Conscientiousness group (β = .041; *p* = .265). We were also interested in whether participants reported stronger GCF the week after they reported higher personality states. Contrary to our assumptions, none of the cross-lagged associations from the weekly personality states to later GCF were significant. Overall, the RI-CLPM findings suggested that participants showed higher state levels in the same week and the week after they reported stronger realization of GCF.

**Figure 4.**
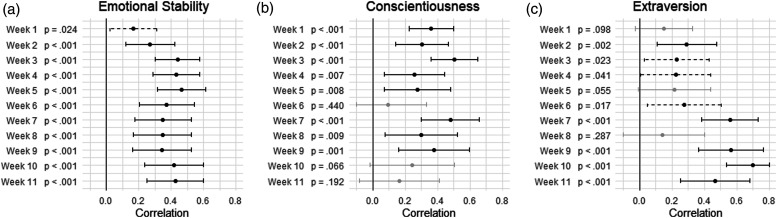
Within-person occasion-specific correlations across weeks and groups. (a) Emotional stability, (b) conscientiousness, and (c) extraversion. *Note*. Presented are the occasion-specific correlations in each week with 95% confidence intervals. Gray lines indicate *p* > .05; dashed lines *p* ≤ .05; solid black lines *p* ≤ .010.

As constraining the within-person occasion-specific correlations (Figure 3(b)) to equality across time increased model misfit for the Conscientiousness and Extraversion group (see Table 3), we also examined whether these correlations differed significantly across time. The correlations at each week are presented in Figure 4. As suggested by the equality constraints, the associations were stable across time in the ES group. The variations in the CO group were unsystematic across time. For the EX group, the associations seemed to be stronger in the latter half of the covered time span. A linear regression with the correlation as independent variable and week as dependent variable suggested an average increase of Δ*r* = .04 per week (*p* = .018).

## Discussion

The main goal of this study was to examine whether a greater realization of the generic change factors (GCF) of strengths, insight, and behavioral practice through a digital intervention is associated with greater changes in weekly personality states. The present results provide multiple insights into potential mechanisms of change in personality change interventions. First, the measurement of the GCF showed a single-factor structure, both at the between-person and within-person levels. This suggests that individuals focused on the GCF simultaneously rather than separately. Second, the weekly states of Emotional Stability, Conscientiousness, Extraversion and levels of GCF increased during the intervention. This is consistent with previously reported increases at the trait level between the beginning and end of the intervention (Stieger et al., 2021) and further indicates that participants also gained more self-insight, the ability to modify behaviors, or access strengths and resources. Third, stronger increases in the generic change factors were associated with stronger increases in personality states during the same week. This finding is initial evidence of within-person coupled change between intervention implementation of GCF and personality states. Fourth, stronger increases in GCF were associated with stronger personality state change in the following week for Emotional Stability and Extraversion—but not for Conscientiousness. In contrast, personality state changes were not associated with an increase in the GCF in the following week. In summary, the present study provides novel findings for the within-person dynamics of personality change during a personality intervention.

### Strengths, insight, and behavioral practice merge into one generic change factor

We examined the factor structure of GCF at both the between-person and within-person levels, as we wanted to examine if people would focus on strengths, insight, and behavioral practice to different degrees, both in comparison to others (e.g., difference in main intervention processes between groups) and across time (e.g., one change factor prioritized per week). This is consistent with recent calls for future research on GCF to focus not only on differences between individuals but also on variability within individuals so that GCF can be linked to outcome processes (Cuijpers et al., 2019).

Despite a better model fit of the three-factor model of GCF compared to the one-factor model, the strong between- and within-person factor loadings and correlations supported one general factor at both levels. This suggests that the intervention simultaneously promoted strengths, insight, and behavioral practice, not only over the entire duration of the intervention, but also on a weekly basis. One obvious reason for this finding might be that the intervention addressed all factors simultaneously, using multiple activities and tasks to maximize the intervention effect (Stieger et al., 2018, 2021). Due to this *broad* and *comprehensive* intervention approach, it might have been difficult to differentiate between the different generic change factors. Indeed, previous work from psychotherapy literature suggests similar findings regarding the structure of GCF (Flückiger et al., 2010), indicating that the various retrospectively assessed, patient-evaluated change factors are highly interrelated (e.g., Finsrud et al., 2022; Wampold & Flückiger, 2023). It could also be because, due to the short measurement with three items per subscale, the scale does not differentiate between GCF as could be the case with longer scales.

The present results on the structure of the GCF raise interesting questions for future research. First, are people able to differentiate between different change factors? Measures that address key aspects of the interventional process in face-to-face sessions, such as realization of strengths, typically assess the realization of GCF immediately after a therapy session to check what has been implemented in that particular session (Flückiger et al., 2010; Krampen & Wald, 2001; Trösken, 2006). However, in the present study, the temporal focus of the assessment referred to the *last week*. Therefore, it is possible that temporal effects were confounded due to daily intervention activities and tasks (e.g., more behavioral practice on one day and more insight on another day). Second, are the items (Table 2) measuring the GCF too similar because they all relate to some degree to (self-) perception or self-knowledge? Although the face validity of the items suggests different concepts, it is possible that processes of self-perception and self-knowledge have contributed to the single-factor structure of GCF.

### Changes in weekly personality states and generic change factors

We found within-person increases in the states of Emotional Stability, Conscientiousness, and Extraversion in the corresponding change goal group with similar rates of change. Those who wanted to become more emotionally stable or extraverted on average showed a steady increase in Emotional Stability and Extraversion states from one week to the next, while the Conscientiousness group tended to show a faster increase at the beginning of the intervention and a slower increase toward the end of the intervention. Overall, these results provide important additional evidence for the effectiveness of the personality coaching intervention (Stieger et al., 2021), and for intervention efforts to change personality traits in general (e.g., Jackson et al., 2021; Roberts, Luo, et al., 2017; Stieger, Wepfer, et al., 2020).

We also found evidence of an average within-person increase in the measure of generic change factors across time, with the increase being initially faster but then slower in all three groups. The rates of change in the GCF seemed to be similar in all groups. This increase in GCF is important because it suggests that increasingly more supportive, learning, and action-related change factors were realized over the course of the digital intervention, at least in participants’ perceptions of learning more about themselves, exhibiting more (or new) behaviors, and experiencing more support from others and from the digital coach.

Despite an average increase in within-person change trajectories for personality states and GCF, we observed considerable variability in these trajectories, as not every individual changed in a similar way or direction. Indeed, one of the most fundamental observation in intervention science is that people often differ in how they respond to and change due to interventions (e.g., Barkham et al., 2021). The same treatment can have different effects on people in the way change processes occur within people. Evidence of variability in personality state change and change factors was therefore an important prerequisite for linking change processes to personality states.

### The realization of generic change factors promotes weekly personality state change

We tested the assumption that the realization of GCF is partly responsible for the effects of personality change during the time of the intervention in two different ways. First, we examined whether and how within-person changes in the change factors and in personality states are coupled both within and across individuals. On the within-person level, we found significant within-time associations for Conscientiousness, Extraversion, and Emotional Stability. According to effect size interpretation for psychological research (Funder & Ozer, 2019), these effects reflect medium to large effects. The findings indicate that in those weeks in which people showed the strongest state change, they also experienced the strongest change in GCF. The directionality could be that stronger activation of GCF triggered a stronger state change that week, but also that exploring more behaviors, thoughts, or feelings triggered more awareness of own capabilities and strengths. So far, these results provide initial evidence for within-person coupled change of change factors with increases in personality states.

On the between-person level, we found medium to strong correlations of Conscientiousness and Extraversion with the GCF. On one hand, these results could be an indication that generally more conscientious (i.e., the tendency to be self-controlled, responsible to others, hardworking, orderly, and rule abiding; Roberts et al., 2014) or extraverted (i.e., sensitivity to potential rewards in social situations; Lucas et al., 2000; and tendency to experience frequent positive affect; Fleeson et al., 2002) individuals engaged more with the intervention and thus achieved higher GCF levels overall. On the other hand, it might also be that the Extraversion and Conscientiousness state levels were highest for those persons who achieved the strongest GCF levels during the intervention. However, this assumption is not supported by the Emotional Stability findings, for which we did not find a statistically significant between-person association. As expected, weeks in which participants increased in their Emotional Stability were also characterized by higher evaluated GCF. But participants with the highest Emotional Stability level during the intervention did not show highest evaluations on the change factors overall. One interpretation of this finding could be that individuals with high Emotional Stability tend to avoid strong emotional states in both directions (positive and negative), which could also manifest in more balanced, less euphoric ratings of the change factors (e.g., Ringwald et al., 2023). Alternatively, our result may suggest that coupling with Emotional Stability primarily reflects a within-person process or is simply a random finding. Research is needed to replicate this finding.

Second, we examined cross-lagged effects of GCF and personality states from one week to the next to investigate the directionality of the coupled change processes. Perhaps most intriguing about the present results is that within-person increases in the GCF were moderately associated with subsequent increases in Extraversion and Emotional Stability during the following week. We did not find this for Conscientiousness. However, the cross-lags could be constrained to equality across groups, suggesting that the effects of the GCF on states in the next week might be generalizable across groups. This suggests that, in addition to moderate to strong within-time correlations, the GCF appear to trigger further state changes beyond the current week. In contrast, we did not find effects of within-person increases in the state levels on subsequent change factors the week after. It may not be surprising that state changes do not affect subsequent GCF changes, but the impact may be more immediate (i.e., in the same week). In addition, it could be that the better the goals are achieved during the course of the intervention, the less pressure there is to engage in self-change efforts.

Although cross-lagged effects do not provide evidence for causal processes in a strict sense, as is the case with experiments, the present results provide initial support (with the exception of Conscientiousness) for the notion that the realization of GCF is partly responsible for the effects of the personality change intervention (Allemand & Flückiger, 2017, 2022). Overall, the present findings contribute to a better understanding of the dynamic processes underlying personality development as discussed in conceptual accounts of personality development (e.g., Wrzus & Roberts, 2017). Based on the TESSERA framework (Wrzus & Roberts, 2017) and the present findings, we therefore assume that the intervention with all its activities and tasks triggered both reflective and associative processes that contributed to the observed changes in personality states.

### Implications, limitations, and future directions

The present findings have major implications. While intervention theories focus in part on describing specific pathways of change, the present study may indicate that a critical point in interventional change is that the various GCF are aligned to each other. That is, insight, practiced behaviors, and support may be most effective when they are *coordinated* (e.g., Flückiger et al., 2022). Another practical implication of this finding might be that individuals are more likely to assess their engagement in the intervention from a holistic perspective and that this assessment corresponds to a more optimistic expectation of the intervention (Gallagher et al., 2020; Wampold & Imel, 2015). Overall, the realization of change factors may increase the likelihood of subsequent change in personality states, especially for individuals who wish to cultivate affective traits, that is, Emotional Stability and Extraversion.

The present study has some limitations that may inform future directions. First, the measure of GCF was intended to capture strengths, insight, and behavioral practice as generic change factors. However, the empirical data in this study support a single-factor model that includes all items of the three postulated factors. Since support, learning, and action-related general change factors cannot be reduced to just the three factors studied, but include many additional factors (Cuijpers et al., 2019; Lambert & Ogles, 2004), it would be valuable to study additional GCF as potential mechanisms of change. For instance, discrepancy awareness is an important candidate that was not included in the measure of change factors. Future research is needed to examine discrepancy awareness over time. Awareness of discrepancy as a support factor may be temporally before the other change factors, and the magnitude of discrepancy should decrease as the intervention becomes more effective. Moreover, another candidate is working alliance (i.e., the collaborative quality between clients and professionals). Meta-analytic work reports a robust positive association between working alliance and treatment outcomes of psychotherapy and coaching (Flückiger et al., 2018; Grassmann et al., 2020). There is also preliminary evidence that individuals can develop an affective bond with chatbots (Nißen et al., 2022). Consequently, digital personality change interventions with chatbots may consider working alliance as a change factor.

Second, the process assessments of personality states and GCF were based only on self-reports. It would be valuable to supplement the self-reports with other assessment methods, such as observer reports, as was done for the outcome assessment of the personality change intervention (Olaru et al., 2022; Stieger et al., 2021). Measures that address GCF in clinical and psychotherapeutic settings typically supplement self-reports with reports from therapists (Flückiger et al., 2010; Krampen & Wald, 2001). However, this was not possible in the present study because a chatbot-based digital coach was used. In addition to the demanding nature of requesting weekly observer reports from informants such as friends and family members, it is unlikely that they will be able to observe participants for a sufficient length of time over the course of a week to provide reliable measures of personality states. Future research, however, could combine digital interventions with face-to-face interventions and include observer reports from the coaches or psychologists, or alternatively include some naturalistic behavioral observations through mobile sensing approaches (e.g., Beierle et al., 2024; Mehl, 2017; Rüegger et al., 2020).

Third, a criticism related to self-reported changes is that participants may have reported changes based on expectations regarding the intervention goals or wishful thinking. The impact of demand effects is not limited to personality change interventions, but is a challenge commonly faced in psychotherapy, counseling, and coaching. It is difficult to completely eliminate these effects. However, it is important that future studies of personality change interventions consider the potential impact of demand characteristics and explicitly control for them in the research design (e.g., double-blind design and implicit measures).

Fourth, a valuable addition to future personality intervention research is to examine whether the changes achieved during an intervention can be achieved over long-term periods. A recent one-year follow-up of the PEACH intervention has shown that the achieved personality changes remained stable (for those who wanted to increase in extraversion and conscientiousness) or even changed further (for those who wanted to decrease in neuroticism) (Stieger, Flückiger, & Allemand, 2023). However, it is important to consider attrition effects, as participants who completed the one-year follow-up were more open to experience, less neurotic, more agreeable, and conscientious than those who did not complete the one-year follow-up.

Fifth, another valuable addition to future personality intervention research is to evaluate potential consequences of the changes in behaviors and change factors. Initial work suggests that reaching personality change goals can improve, or at least be associated with subjective well-being (Hudson & Fraley, 2016) or physical activity (Stieger, Allemand, & Lachman, 2023). A recent study showed that the present personality intervention resulted in an increase in life satisfaction directly after intervention and in the 3-month follow-up (Olaru et al., 2023). An open question is whether these changes can already be observed during the first weeks of the intervention or take more time to manifest. Studies asking participants to behave more extraverted during short durations of time (e.g., one week) generally also found improvements in positive affect (Margolis & Lyubomirsky, 2020; van Allen et al., 2021), but also increases in negative affect and tiredness for more introverted participants (Jacques-Hamilton et al., 2019). One assumption could be that potential negative side-effects of “acting out-of-character” (Kuijpers et al., 2022) dissipate as behaviors become more habitual during the course of the intervention.

Finally, while the sample size is impressive for an intervention study, it is a relatively culturally homogeneous sample of rather younger Swiss adults recruited primarily in educational settings; thus, work is needed to understand the generalizability of the findings in more heterogeneous contexts and using additional cohorts.

### Conclusion

The present research makes four important contributions to the study of personality trait change through psychological interventions. First, it shows that the potential generic change mechanisms of strengths, insight, and behavioral practice tend to merge into a single-factor model of change factors within and between individuals. Second, it provides further evidence for the effectiveness of a digital intervention (Stieger et al., 2021) by showing within-person increases in weekly personality states, and in generic change factors during the intervention. Third, it shows that within-person changes in personality states and change factors are coupled over time. Finally, it provides initial support for the claim that the realization of strengths, insight, and behavioral practice partly explain the effects of the personality change intervention. Most interestingly, within-person increases in generic change factors were prospectively associated with subsequent increases in Extraversion and Emotional Stability during the following week.

## Notes

1. Previous studies used longitudinal *outcome data* from the digital personality change intervention trial to examine the effects of the digital intervention at the trait domain level (Stieger et al., 2021), the facet and nuance levels (Olaru et al., 2022), the one-year maintenance effects (Stieger, Flückiger, & Allemand, 2023), associations between changes in traits and self-esteem (Allemand et al., 2024) and life satisfaction (Olaru et al., 2023). One study used *pretest data* to examine predictors of personality change goals (Stieger, Eck, et al., 2020). Two studies used the one-week *ambulatory assessment data* before the intervention phase to examine associations between personality states and situation cues (Lindner et al., 2023), and associations between personality states and smartphone sensing (Rüegger et al., 2020). The intensive longitudinal *process data* on weekly personality states and GCF are unique to this study and have not been previously published.

2. As a further safeguard against possible psychological and technical problems, it was possible to contact the multidisciplinary study team directly via the app. The team also included a psychotherapist (the last author). During the intervention phase and afterward, no individuals reported psychological problems as a result of the intervention. However, the team was sometimes contacted about technical problems with the app.

3. We also ran the multi-level confirmatory factor analysis including participants from the excluded change goal groups (e.g., increase Open-mindedness; for a total of *N* = 874 vs. 679) (see OSF Table S3). The results were highly similar to the one’s presented in Table 5 (see OSF Table S1).

## References

[bibr1-08902070231225803] Allan J. Leeson P. De Fruyt F. Martin S. (2018). Application of a 10 week coaching program designed to facilitate volitional personality change: Overall effects on personality and the impact of targeting. International Journal of Evidence Based Coaching and Mentoring, 16(1), 80–94. 10.24384/000470

[bibr2-08902070231225803] Allemand M. Flückiger C. (2017). Changing personality traits: Some considerations from psychotherapy process-outcome research for intervention efforts on intentional personality change. Journal of Psychotherapy Integration, 27(4), 476–494. 10.1037/int0000094

[bibr100-08902070231225803] Allemand M. Flückiger C. (2020). Different routes, same effects: Managing unresolved interpersonal transgressions in old age. Journal of Gerontopsychology and Geriatric Psychiatry, 33(4), 223–234. 10.1024/1662-9647/a000237

[bibr3-08902070231225803] Allemand M. Flückiger C. (2022). Personality change through digital-coaching interventions. Current Directions in Psychological Science, 31(1), 41–48. 10.1177/09637214211067782

[bibr4-08902070231225803] Allemand M. Gmür B. Flückiger C. (2022). Does extraversion increase following a three‐hour flirt training? Exploring two training routes. Scandinavian Journal of Psychology, 63(3), 265–274. 10.1111/sjop.12803 35301728 PMC9313810

[bibr5-08902070231225803] Allemand M. Olaru G. Stieger M. Flückiger C. (2024). Intervention-related correlated change between personality traits and self-esteem. Consulting Psychology Journal. https://doi/10.1037/cpb0000266

[bibr6-08902070231225803] Allemand M. Stieger M. (2024). Digital coaching to promote and manage change. In Taku K. Shackelford T. K. (Eds.), The Routledge international handbook of changes in human perceptions and behaviors. Routledge.

[bibr7-08902070231225803] Armstrong L. Rimes K. A. (2016). Mindfulness-based cognitive therapy for neuroticism (stress vulnerability): A pilot randomized study. Behavior Therapy, 47(3), 287–298. 10.1016/j.beth.2015.12.005 27157024

[bibr8-08902070231225803] Barkham M. Lutz W. Castonguay L. G. (Eds.). (2021). Bergin and Garfield’s handbook of psychotherapy and behavior change (7th ed.), Wiley. 10.1055/a-1686-4682 34979586

[bibr9-08902070231225803] Baumert A. Schmitt M. Perugini M. Johnson W. Blum G. Borkenau P. Costantini G. Denissen J. J. A. Fleeson W. Grafton B. Jayawickreme E. Kurzius E. MacLeod C. Miller L. C. Read S. J. Roberts B. Robinson M. D. Wood D. Wrzus C. (2017). Integrating personality structure, personality process, and personality development. European Journal of Personality, 31(5), 503–528. 10.1002/per.2115

[bibr10-08902070231225803] Beierle F. Matz S. C. Allemand M. (2024). Mobile sensing in personality science. In Mehl M. R. Wrzus C. Eid M. Harari G. Ebner-Priemer U. (Eds.), Mobile sensing in psychology: Methods and applications. Guilford Press.

[bibr11-08902070231225803] Bentler P. M. (1990). Comparative fit indexes in structural models. Psychological Bulletin, 107(2), 238–246. 10.1037/0033-2909.107.2.238 2320703

[bibr12-08902070231225803] Bleidorn W. Schwaba T. Zheng A. Hopwood C. J. Sosa S. S. Roberts B. W. Briley D. A. (2022). Personality stability and change: A meta-analysis of longitudinal studies. Psychological Bulletin, 148(7-8), 588–619. 10.1037/bul0000365 35834197

[bibr13-08902070231225803] Carver C. S. Scheier M. F. (1998). On the self-regulation of behavior. Cambridge University Press.

[bibr14-08902070231225803] Castonguay L. G. Eubanks C. F. Goldfried M. R. Muran J. C. Lutz W. (2015). Research on psychotherapy integration: Building on the past, looking to the future. Psychotherapy Research, 25(3), 365–382. 10.1080/10503307.2015.1014010 25800531

[bibr15-08902070231225803] Chapman B. P. Hampson S. Clarkin J. (2014). Personality-informed interventions for healthy aging: Conclusions from a National Institute on Aging work group. Developmental Psychology, 50(5), 1426–1441. 10.1037/a0034135 23978300 PMC3940665

[bibr16-08902070231225803] Cheavens J. S. Strunk D. R. Lazarus S. A. Goldstein L. A. (2012). The compensation and capitalization models: A test of two approaches to individualizing the treatment of depression. Behaviour Research and Therapy, 50(11), 699–706. 10.1016/j.brat.2012.08.002 22982085

[bibr17-08902070231225803] Cuijpers P. Reijnders M. Huibers M. J. H. (2019). The role of common factors in psychotherapy outcomes. Annual Review of Clinical Psychology, 15, 207–231. 10.1146/annurev-clinpsy-050718-095424 30550721

[bibr18-08902070231225803] Curran P. J. Howard A. L. Bainter S. A. Lane S. T. McGinley J. S. (2014). The separation of between-person and within-person components of individual change over time: A latent curve model with structured residuals. Journal of Consulting and Clinical Psychology, 82(5), 879–894. 10.1037/a0035297 24364798 PMC4067471

[bibr19-08902070231225803] Danner D. Rammstedt B. Bluemke M. Lechner C. Berres S. Knopf T. Soto C. J. John O. P. (2019). Das Big Five Inventar 2: Validierung eines Persönlichkeitsinventars zur Erfassung von 5 Persönlichkeitsdomänen und 15 Facetten [The German Big Five Inventory 2: Measuring five personality domains and 15 facets]. Diagnostica, 65(3), 121–132. 10.1026/0012-1924/a000218

[bibr20-08902070231225803] De Fruyt F. Van Leeuwen K. Bagby R. M. Rolland J.-P. Rouillon F. (2006). Assessing and interpreting personality change and continuity in patients treated for major depression. Psychological Assessment, 18(1), 71–80. 10.1037/1040-3590.18.1.71 16594814

[bibr21-08902070231225803] Eldridge F. Dembkowski S. (2013). Behavioral coaching. In Passmore J. Peterson D. B. Freire T. (Eds.), The Wiley-Blackwell handbook of the psychology of coaching and mentoring (pp. 298–318), Wiley Blackwell.

[bibr22-08902070231225803] Finsrud I. Nissen-Lie H. A. Vrabel K. Høstmælingen A. Wampold B. E. Ulvenes P. G. (2022). It’s the therapist and the treatment: The structure of common therapeutic relationship factors. Psychotherapy Research, 32(2), 139–150. 10.1080/10503307.2021.1916640 33938407

[bibr23-08902070231225803] Fleeson W. Malanos A. B. Achille N. M. (2002). An intraindividual process approach to the relationship between extraversion and positive affect: Is acting extraverted as “good” as being extraverted? Journal of Personality and Social Psychology, 83(6), 1409–1422. 10.1037/0022-3514.83.6.1409 12500821

[bibr24-08902070231225803] Flückiger C. Del Re A. C. Wampold B. E. Horvath A. O. (2018). The alliance in adult psychotherapy: A meta-analytic synthesis. Psychotherapy, 55(4), 316–340. 10.1037/pst0000172 29792475

[bibr25-08902070231225803] Flückiger C. Horvath A. O. Brandt H. (2022). The evolution of patients’ concept of the alliance and its relation to outcome: A dynamic latent-class structural equation modeling approach. Journal of Counseling Psychology, 69(1), 51–62. 10.1037/cou0000555 34197151

[bibr26-08902070231225803] Flückiger C. Munder T. Del Re A. C. Solomonov N. (2023). Strength-based methods - a narrative review and comparative multilevel meta-analysis of positive interventions in clinical settings. Psychotherapy Research, 33(7), 856–872. 10.1080/10503307.2023.2181718 36863015 PMC10440292

[bibr27-08902070231225803] Flückiger C. Regli D. Zwahlen D. Hostettler S. Caspar F. (2010). Der Berner Patienten- und Therapeutenstundenbogen 2000: Ein Instrument zur Erfassung von Therapieprozessen [The Bern Post Session Report 2000, patient and therapist versions: Measuring psychotherapeutic processes]. Zeitschrift für Klinische Psychologie und Psychotherapie, 39(2), 71–79. 10.1026/1616-3443/a000015

[bibr28-08902070231225803] Flückiger C. Vîslă A. Wolfer C. Hilpert P. Zinbarg R. E. Lutz W. grosse Holtforth M. Allemand M. (2021). Exploring change in cognitive-behavioral therapy for generalized anxiety disorder—a two-arms ABAB crossed-therapist randomized clinical implementation trial. Journal of Consulting and Clinical Psychology, 89(5), 454–468. 10.1037/ccp0000639 33829819

[bibr29-08902070231225803] Funder D. C. Ozer D. J. (2019). Evaluating effect size in psychological research: Sense and nonsense. Advances in Methods and Practices in Psychological Science, 2(2), 156–168. 10.1177/2515245919847202

[bibr30-08902070231225803] Gallagher M. W. Long L. J. Richardson A. D'Souza J. Boswell J. F. Farchione T. J. Barlow D. H. (2020). Examining hope as a transdiagnostic mechanism of change across anxiety disorders and CBT treatment protocols. Behavior Therapy, 51(1), 190–202. 10.1016/j.beth.2019.06.001 32005336 PMC7000132

[bibr31-08902070231225803] Gómez-Penedo J. M. Babl A. Dyresen A. Fernández-Álvarez J. Flückiger C. Grosse Holtforth M. (2023). Problem mastery and motivational clarification as mechanisms of change in cognitive-behavioral therapy for depression: Secondary analysis of a randomized controlled trial. Behaviour Research and Therapy, 167, 104343. 10.1016/j.brat.2023.104343 37307656

[bibr32-08902070231225803] Graham E. K. Weston S. J. Gerstorf D. Yoneda T. B. Booth T. Beam C. R. Petkus A. J. Drewelies J. Hall A. N. Bastarache E. D. Estabrook R. Katz M. J. Turiano N. A. Lindenberger U. Smith J. Wagner G. G. Pedersen N. L. Allemand M. Spiro A. Mroczek D. K. (2020). Trajectories of big five personality traits: A coordinated analysis of 16 longitudinal samples. European Journal of Personality, 34(3), 301–321. 10.1002/per.2259 33564207 PMC7869960

[bibr33-08902070231225803] Grawe K. (2004). Psychological therapy, Hogrefe & Huber Publishers.

[bibr34-08902070231225803] Graßmann C. Schölmerich F. Schermuly C. C. (2020). The relationship between working alliance and client outcomes in coaching: A meta-analysis. Human Relations, 73(1), 35–58. 10.1177/0018726718819725

[bibr102-08902070231225803] Grawe K. Donati R. Bernbauer F. (1994). Psychotherapie im Wandel. Von der Konfession zur Profession. [Psychotherapy in transition. From denomination to profession]. Hogrefe.

[bibr35-08902070231225803] Grosse Holtforth M. Flückiger C. (2012). The stream of corrective experiences in action: Big bang and constant dripping. In Castonguay L. G. Hill C. E. (Eds.), Transformation in psychotherapy: Corrective experiences across cognitive behavioral, humanistic, and psychodynamic approaches (pp. 317–333). American Psychological Association. 10.1037/13747-015

[bibr36-08902070231225803] Grosz M. P. Lemp J. M. Rammstedt B. Lechner C. M. (2022). Personality change through arts education: A review and call for further research. Perspectives on Psychological Science, 17(2), 360–384. 10.1177/1745691621991852 34283673 PMC8902031

[bibr37-08902070231225803] Hamaker E. L. Kuiper R. M. Grasman R. P. (2015). A critique of the cross-lagged panel model. Psychological Methods, 20(1), 102–116. 10.1037/a0038889 25822208

[bibr38-08902070231225803] Hennecke M. Bleidorn W. Denissen J. J. A. Wood D. (2014). A three‐part framework for self‐regulated personality development across adulthood. European Journal of Personality, 28(3), 289–299. 10.1002/per.1945

[bibr39-08902070231225803] Hu L. T. Bentler P. M. (1999). Cutoff criteria for fit indexes in covariance structure analysis: Conventional criteria versus new alternatives. Structural Equation Modeling: A Multidisciplinary Journal, 6(1), 1–55. 10.1080/10705519909540118

[bibr40-08902070231225803] Hudson N. W. (2023). Lighten the darkness: Personality interventions targeting agreeableness also reduce participants’ levels of the dark triad. Journal of Personality, 91(4), 901–916. 10.1111/jopy.12714 35285028

[bibr41-08902070231225803] Hudson N. W. Briley D. A. Chopik W. J. Derringer J. (2019). You have to follow through: Attaining behavioral change goals predicts volitional personality change. Journal of Personality and Social Psychology, 117(4), 839–857. 10.1037/pspp0000221 30359069

[bibr42-08902070231225803] Hudson N. W. Fraley R. C. (2016). Changing for the better? Longitudinal associations between volitional personality change and psychological well-being. Personality and Social Psychology Bulletin, 42(5), 603–615. 10.1177/0146167216637840 27016068

[bibr43-08902070231225803] Hudson N. W. Fraley R. C. Chopik W. J. Briley D. A. (2020). Change goals robustly predict trait growth: A mega-analysis of a dozen intensive longitudinal studies examining volitional change. Social Psychological and Personality Science, 11(6), 723–732. 10.1177/1948550619878423

[bibr44-08902070231225803] Jackson J. J. Beck E. D. Mike A. (2021). Personality interventions. In John O. P. Robins R. W. (Eds.), Handbook of personality: Theory and research (pp. 793–805), Guilford Press.

[bibr45-08902070231225803] Jackson J. J. Hill P. L. Payne B. R. Roberts B. W. Stine-Morrow E. A. L. (2012). Can an old dog learn (and want to experience) new tricks? Cognitive training increases openness to experience in older adults. Psychology and Aging, 27(2), 286–292. 10.1037/a0025918 22251379 PMC3330146

[bibr46-08902070231225803] Jacques-Hamilton R. Sun J. Smillie L. D. (2019). Costs and benefits of acting extraverted: A randomized controlled trial. Journal of Experimental Psychology: General, 148(9), 1538–1556. 10.1037/xge0000516 30489119

[bibr47-08902070231225803] Javaras K. N. Williams M. Baskin-Sommers A. R. (2019). Psychological interventions potentially useful for increasing conscientiousness. Personality Disorders: Theory, Research, and Treatment, 10(1), 13–24. 10.1037/per0000267 30604980

[bibr48-08902070231225803] Krampen G. Wald B. (2001). Kurzinstrumente für die Prozessevaluation und adaptive Indikation in der Allgemeinen und differentiellen Psychotherapie und Beratung [brief instruments for process evaluation and adaptive indication in general and differential psychotherapy and counseling. Diagnostica, 47(1), 43–50. 10.1026//0012-1924.47.1.43

[bibr49-08902070231225803] Kuijpers E. Dirkx I. Wille B. Hofmans J. (2022). A multidimensional approach to acting out of character: How deviating from one’s personality profile relates to resource depletion and affect. Journal of Research in Personality, 97, 104192. 10.1016/j.jrp.2022.104192

[bibr50-08902070231225803] Lambert M. J. Ogles B. M. (2004). The efficacy and effectiveness of psychotherapy. In Lambert M. J. (Ed.), Bergin and Garfield’s handbook of psychotherapy and behavior change (5th ed., pp. 139–193), Wiley.

[bibr51-08902070231225803] Lindner S. Stieger M. Rüegger D. Kowatsch T. Flückiger C. Mehl M. R. Allemand M. (2023). How is variety in daily life related to the expression of personality states? An ambulatory assessment study. European Journal of Personality, . 10.1177/08902070221149593 PMC13029511 41908318

[bibr52-08902070231225803] Lucas R. E. Diener E. Grob A. Suh E. M. Shao L. (2000). Cross-cultural evidence for the fundamental features of extraversion. Journal of Personality and Social Psychology, 79(3), 452–468. 10.1037//0022-3514.79.3.452 10981846

[bibr53-08902070231225803] Lutz W. Castonguay L. G. Lambert M. J. Barkham M. (2021). Traditions and new beginnings: Historical and current perspectives on research in psychotherapy and behavior change. In Barkham M. Lutz W. Castonguay L. G. (Eds.), Bergin and Garfield’s handbook of psychotherapy and behavior change (7th ed., pp. 3–18), Wiley.

[bibr54-08902070231225803] Magidson J. F. Roberts B. W. Collado-Rodriguez A. Lejuez C. W. (2014). Theory-driven intervention for changing personality: Expectancy value theory, behavioral activation, and conscientiousness. Developmental Psychology, 50(5), 1442–1450. 10.1037/a0030583 23106844 PMC3646072

[bibr55-08902070231225803] Margolis S. Lyubomirsky S. (2020). Experimental manipulation of extraverted and introverted behavior and its effects on well-being. Journal of Experimental Psychology: General, 149(4), 719–731. 10.1037/xge0000668 31368759

[bibr56-08902070231225803] Martin L. S. Oades L. G. Caputi P. (2014). A step-wise process of intentional personality change coaching. International Coaching Psychology Review, 9(2), 181–195. 10.53841/bpsicpr.2014.9.2.181

[bibr57-08902070231225803] Massey-Abernathy A. R. Robinson D. N. (2021). Personality promotion: The impact of coaching and behavioral activation on facet level personality change and health outcomes. Current Psychology, 40(12), 5984–5995. 10.1007/s12144-019-00530-4

[bibr58-08902070231225803] Mehl M. R. (2017). The Electronically Activated Recorder (EAR): A method for the naturalistic observation of daily social behavior. Current Directions in Psychological Science, 26(2), 184–190. 10.1177/0963721416680611 28529411 PMC5434514

[bibr59-08902070231225803] Mund M. Nestler S. (2019). Beyond the Cross-Lagged Panel Model: Next-generation statistical tools for analyzing interdependencies across the life course. Advances in Life Course Research, 41, 100249. 10.1016/j.alcr.2018.10.002 36738028

[bibr60-08902070231225803] Muthen L. K. Muthen B. O. (1998-2017), Mplus user’s guide. Muthen and Muthen.

[bibr61-08902070231225803] Nevins B. G. (2021). Applying personality-informed treatment strategies to clinical practice: A theoretical and practical guide, Routledge/Taylor & Francis Group.

[bibr62-08902070231225803] Nißen M. Rüegger D. Stieger M. Flückiger C. Allemand M. Wangenheim F. Kowatsch T. (2022). The effects of health care chatbot personas with different social roles on the client-chatbot bond and usage intentions: Development of a design codebook and web-based study. Journal of Medical Internet Research*, *24(4), Article e32630. 10.2196/32630 PMC9096656 35475761

[bibr63-08902070231225803] Norcross J. C. Goldfried M. R. (Eds.), (2019). Handbook of psychotherapy integration. (3rd ed.), Oxford University Press. 10.1093/med-psych/9780190690465.001.0001

[bibr64-08902070231225803] Olaru G. Allemand M. (2022). Correlated personality change across time and age. European Journal of Personality, 36(5), 729–749. 10.1177/08902070211014054

[bibr65-08902070231225803] Olaru G. Stieger M. Rüegger D. Kowatsch T. Flückiger C. Roberts B. W. Allemand M. (2022). Personality change through a digital-coaching intervention: Using measurement invariance testing to distinguish between trait domain, facet, and nuance change. European Journal of Personality, 089020702211450. 10.1177/08902070221145088 PMC13021088 41908316

[bibr66-08902070231225803] Olaru G. van Sheppingen M. A. Stieger M. Kowatsch T. Flückiger C. Allemand M. (2023). The effects of a personality intervention on satisfaction in ten domains of life: Evidence for increases and correlated change with personality traits. Journal of Personality and Social Psychology, 125(4), 902–924. 10.1037/pspp0000474 37498689

[bibr101-08902070231225803] Orlinsky D. E. Grawe K. Parks B. K. (1994). Process and outcome in psychotherapy: Noch einmal. In Bergin A. E. Garfield S. L. (Eds.), Handbook of psychotherapy and behavior change (pp. 270–376). John Wiley & Sons.

[bibr67-08902070231225803] Orth U. Clark D. A. Donnellan M. B. Robins R. W. (2021). Testing prospective effects in longitudinal research: Comparing seven competing cross-lagged models. Journal of Personality and Social Psychology, 120(4), 1013–1034. 10.1037/pspp0000358 32730068 PMC7854859

[bibr68-08902070231225803] Orth U. Meier L. L. Bühler J. L. Dapp L. C. Krauss S. Messerli D. Robins R. W. (2022). Effect size guidelines for cross-lagged effects. Psychological Methods. 10.1037/met0000499 35737548

[bibr69-08902070231225803] Palmer S. Williams H. (2013). Cognitive behavioral approaches. In Passmore J. Peterson D. B. Freire T. (Eds.), The Wiley-Blackwell handbook of the psychology of coaching and mentoring (pp. 319–338), Wiley Blackwell.

[bibr70-08902070231225803] R Core Team . (2022). R: A language and environment for statistical computing: R Foundation for Statistical Computing. URL. https://www.R-project.org/

[bibr71-08902070231225803] Rebele R. W. Koval P. Smillie L. D. (2021). Personality-informed intervention design: Examining how trait regulation can inform efforts to change behavior. European Journal of Personality, 35(4), 623–645. 10.1177/08902070211016251

[bibr72-08902070231225803] Revelle W. (2022). psych: Procedures for personality and psychological research: Northwestern University. https://CRAN.R-project.org/package=psych_Version=2.2.5

[bibr73-08902070231225803] Ringwald W. R. Hallquist M. N. Dombrovski A. Y. Wright A. G. C. (2023). Personality (dys)function and general instability. Clinical Psychological Science, 11(1), 106–120. 10.1177/21677026221083859 36844787 PMC9949732

[bibr74-08902070231225803] Roberts B. W. Hill P. L. Davis J. P. (2017). How to change conscientiousness: The Sociogenomic Trait Intervention Model. Personality Disorders: Theory, Research, and Treatment, 8(3), 199–205. 10.1037/per0000242 29120219

[bibr75-08902070231225803] Roberts B. W. Lejuez C. Krueger R. F. Richards J. M. Hill P. L. (2014). What is conscientiousness and how can it be assessed? Developmental Psychology, 50(5), 1315–1330. 10.1037/a0031109 23276130

[bibr76-08902070231225803] Roberts B. W. Luo J. Briley D. A. Chow P. I. Su R. Hill P. L. (2017). A systematic review of personality trait change through intervention. Psychological Bulletin, 143(2), 117–141. 10.1037/bul0000088 28054797

[bibr77-08902070231225803] Roberts B. W. Walton K. E. Viechtbauer W. (2006). Patterns of mean-level change in personality traits across the life course: A meta-analysis of longitudinal studies. Psychological Bulletin, 132(1), 1–25. 10.1037/0033-2909.132.1.1 16435954

[bibr78-08902070231225803] Rosseel Y. (2012). Lavaan: An*R*Package for structural equation modeling. Journal of Statistical Software, 48(2), 1–36. 10.18637/jss.v048.i02

[bibr79-08902070231225803] Rüegger D. Stieger M. Nißen M. Allemand M. Fleisch E. Kowatsch T. (2020). How are personality states associated with smartphone data? European Journal of Personality, 34(5), 687–713. 10.1002/per.2309

[bibr80-08902070231225803] Sauer-Zavala S. Fournier J. C. Jarvi Steele S. Woods B. K. Wang M. Farchione T. J. Barlow D. H. (2021). Does the unified protocol really change neuroticism? Results from a randomized trial. Psychological Medicine, 51(14), 2378–2387. 10.1017/S0033291720000975 32312357 PMC7678331

[bibr81-08902070231225803] Sauer-Zavala S. Wilner J. G. Barlow D. H. (2017). Addressing neuroticism in psychological treatment. Personality Disorders: Theory, Research, and Treatment, 8(3), 191–198. 10.1037/per0000224 29120218

[bibr82-08902070231225803] Schulte D. (2005). Messung der Therapieerwartung und Therapieevaluation von Patienten (PA-TEV) [Measurement of patients‘ therapy expectation and therapy evaluation]. Zeitschrift für Klinische Psychologie und Psychotherapie, 34(3), 176–188.

[bibr83-08902070231225803] Soto C. J. John O. P. (2017a). The next Big Five Inventory (BFI-2): Developing and assessing a hierarchical model with 15 facets to enhance bandwidth, fidelity, and predictive power. Journal of Personality and Social Psychology, 113(1), 117–143. 10.1037/pspp0000096 27055049

[bibr84-08902070231225803] Soto C. J. John O. P. (2017b). Short and extra-short forms of the Big Five Inventory–2: The BFI-2-S and BFI-2-XS. Journal of Research in Personality, 68, 69–81. 10.1016/j.jrp.2017.02.004

[bibr85-08902070231225803] Stieger M. Allemand M. Lachman M. E. (2023). Effects of a digital self-control intervention to increase physical activity in middle-aged adults. Journal of Health Psychology, 28(10), 984–996. 10.1177/13591053231166756 37042306 PMC10466994

[bibr86-08902070231225803] Stieger M. Allemand M. Roberts B. W. Davis J. P. (2022). Mindful of personality trait change: Are treatment effects on personality trait change ephemeral and attributable to changes in states? Journal of Personality, 90(3), 375–392. 10.1111/jopy.12672 34486730

[bibr87-08902070231225803] Stieger M. Eck M. Rüegger D. Kowatsch T. Flückiger C. Allemand M. (2020). Who wants to become more conscientious, more extraverted, or less neurotic with the help of a digital intervention? Journal of Research in Personality, 87, 103983. 10.1016/j.jrp.2020.103983

[bibr88-08902070231225803] Stieger M. Flückiger C. Allemand M. (2023). One year later: Longer-term maintenance effects of a digital intervention to change personality traits. Journal of Personality. 10.1111/jopy.12898 37929333

[bibr89-08902070231225803] Stieger M. Flückiger C. Rüegger D. Kowatsch T. Roberts B. W. Allemand M. (2021). Changing personality traits with the help of a digital personality change intervention. Proceedings of the National Academy of Sciences of the United States of America*, *118(8), Article e2017548118. 10.1073/pnas.2017548118 PMC7923371 33558417

[bibr90-08902070231225803] Stieger M. Nißen M. Rüegger D. Kowatsch T. Flückiger C. Allemand M. (2018). PEACH, a smartphone-and conversational agent-based coaching intervention for intentional personality change: Study protocol of a randomized, wait-list controlled trial. BMC Psychology, 6(1), 1–15. 10.1186/s40359-018-0257-9 30180880 PMC6123904

[bibr91-08902070231225803] Stieger M. Wepfer S. Rüegger D. Kowatsch T. Roberts B. W. Allemand M. (2020). Becoming more conscientious or more open to experience? Effects of a two-week smartphone-based intervention for personality change. European Journal of Personality, 34(3), 345–366. 10.1002/per.2267

[bibr92-08902070231225803] Tang T. Z. DeRubeis R. J. Hollon S. D. Amsterdam J. Shelton R. Schalet B. (2009). Personality change during depression treatment: A placebo-controlled trial. Archives of General Psychiatry, 66(12), 1322–1330. 10.1001/archgenpsychiatry.2009.166 19996037 PMC2799251

[bibr93-08902070231225803] Trösken A. (2016). BRI – Berner Ressourceninventar [BRI – The Bern ressource inventory]. In Geue K. Strauß B. Brähler E. Hrsg (Eds.), Diagnostische Verfahren in der Psychotherapie (3rd ed., pp. 81–85). Hogrefe.

[bibr94-08902070231225803] van Allen Z. M. Walker D. L. Streiner T. Zelenski J. M. (2021). Enacted extraversion as a well-being enhancing strategy in everyday life: Testing across three, week-long interventions. Collabra: Psychology, 7(1), 29931. 10.1525/collabra.29931

[bibr95-08902070231225803] Wampold B. E. Flückiger C. (2023). The alliance in mental health care: Conceptualization, evidence and clinical applications. World Psychiatry: Official Journal of the World Psychiatric Association (WPA), 22(1), 25–41. 10.1002/wps.21035 36640398 PMC9840508

[bibr96-08902070231225803] Wampold B. E. Imel Z. E. (2015). The great psychotherapy debate: The evidence for what makes psychotherapy work (2nd ed.), Routledge/Taylor & Francis Group.

[bibr97-08902070231225803] Wickham H. (2016). ggplot2: Elegant graphics for data analysis: Springer-Verlag.

[bibr98-08902070231225803] Wickham H. Miller E. Smith D. (2022). haven: Import and export “SPSS”, “Stata” and “SAS” files. R package version 2.5.0. https://CRAN.R-project.org/package=haven

[bibr99-08902070231225803] Wrzus C. Roberts B. W. (2017). Processes of personality development in adulthood: The TESSERA framework. Personality and Social Psychology Review, 21(3), 253–277. 10.1177/1088868316652279 27260302

